# Engaging Children and Young People in Pediatric Palliative Care Research: A Scoping Review Protocol of Patient and Public Involvement Practices

**DOI:** 10.12688/hrbopenres.14236.1

**Published:** 2025-09-23

**Authors:** Aima Molati, Razieh Safarifard, Emma Nicholson, Mary Nevin, Veronica Lambert

**Affiliations:** 1 School of Nursing, Psychotherapy and Community Health, Faculty of Science and Health, Dublin City University - Glasnevin Campus, Glasnevin, County Dublin, D09 V209, Ireland; 2 School of Psychology, Dublin City University - Glasnevin Campus, Glasnevin, County Dublin, D09 V209, Ireland

**Keywords:** Paediatric Palliative Care, Patient and Public Involvement, Children and Young People, Youth Engagement, Participatory research, Scoping review protocol

## Abstract

**Background:**

In paediatric palliative care (PPC) research, meaningful engagement of children and young people (CYP) remains underdeveloped. Despite widespread endorsement of patient and public involvement (PPI) for its ability to enhance research relevance, ethics, and overall impact, current studies provide limited insight into how CYP are involved – specifically, the types, levels (e.g., consultation, collaboration, child-led), and stages (from priority setting to dissemination). This scoping review aims to systematically map existing literature on the involvement of CYP (aged 4–24 years) as active PPI contributors in PPC research. We will identify and categorize reported outcomes and impacts, explore ethical, practical, and emotional considerations, and determine the key factors that enable or hinder meaningful youth participation.

**Methods:**

The scoping review will adhere to the Joanna Briggs Institute (JBI) methodology. A comprehensive search will be conducted across Embase, CINAHL, PubMed, Scopus, and the Cochrane Library. The search will identify English-language empirical and methodological studies detailing CYP involvement at any stage of the research cycle within PPC. Two independent reviewers will screen titles, abstracts, and full texts, with a third reviewer resolving any discrepancies. Extracted data will be descriptively mapped using a structured extraction table.

**Conclusion:**

This scoping review will provide a comprehensive synthesis of CYP involvement as PPI contributors in PPC research. It will illuminate effective engagement practices, highlight common challenges, and identify critical knowledge gaps. The insights generated from this review will inform the development of more inclusive, ethically grounded, and developmentally appropriate models for involving children and young people as true partners in future palliative care research.

## Introduction

Over the last two decades, paediatric palliative care (PPC) has evolved into a distinct and holistic field, dedicated to addressing the complex physical, emotional, social, and spiritual needs of children and young people (CYP) across their illness trajectory ( Fraser *et al*., 2021; World Health Organization ( WHO, 2023). In parallel, patient and public involvement (PPI) has emerged as a cornerstone of high-quality health research enhancing its relevance, ethics, and overall impact ( Brett *et al*., 2014; Chambers *et al*., 2019; INVOLVE, 2012). The World Health Organisation (WHO) defines PPC as the active, total care of the child’s body, mind, and spirit, beginning at diagnosis and continuing regardless of whether the child is receiving curative treatment ( Benini *et al*., 2022). Globally, children constitute approximately 7% of the palliative care population, highlighting the acute need for services specifically tailored to their unique developmental and psychosocial requirements ( Knaul *et al*., 2020).

Meeting the needs of this expanding population demands approaches that are deeply responsive to the lived experiences of CYP. A growing body of work explores how CYP can contribute meaningfully to this field. Involving them as active partners is a participatory approach that ensures palliative care remains relevant to their emotional well-being, sense of autonomy, and daily life, particularly as the boundaries between clinical care and personal experience are inherently intertwined ( Mitchell *et al*., 2021; Taylor *et al*., 2021). Young people bring unique insights shaped by the complexities of living with a serious illness, which can significantly enhance the relevance, sensitivity, and person-centeredness of PPC research. For example, youth perspectives have demonstrably improved the clarity of PPC research materials ( Mitchell *et al*., 2019), and involving children meaningfully is both feasible and valuable across multiple stages of the research lifecycle ( Rouncefield-Swales *et al*., 2021).

Authentic PPI should not be confined to one-off consultations. Instead, it must be embedded as a continuous, co-productive process throughout the research lifecycle. For example, three levels of youth engagement—consultation, collaboration, and child-led participation— illustrate increasing agency and influence ( Lansdown, 2001). Effective PPI with CYP is a reciprocal, relationship-driven process, positioning young participants as co-researchers who genuinely contribute to shaping study design, materials, and outcomes ( Costello & Dorris, 2020; O’Hara *et al*., 2017). In contrast, tokenistic, “tick-box” approaches fail to redistribute real power and can actively erode young people’s trust and engagement.

When engagement is offered through creative formats—such as storytelling, visual methods, or digital platforms like vlogs—young people express strong interest, even in emotionally challenging areas ( Hilverda *et al*., 2025). Such formats stimulate discussion and reflection, especially among adolescents with varying literacy levels ( Hilverda *et al*., 2025). CYP have successfully participated in advisory roles, contributed to research tool design, and supported dissemination efforts, demonstrating their capacity as meaningful co-creators rather than passive respondents ( Hilverda *et al*., 2025; O’Hara *et al*., 2017). Despite the growing imperative, the implementation of meaningful PPI in paediatric palliative care remains fragmented and inconsistently reported ( Holder *et al*., 2024). Several critical challenges impede genuine involvement: Firstly, a key barrier is tokenistic participation, where CYP are often consulted superficially rather than through meaningful, empowering involvement. In PPC research specifically, young people have expressed concerns that their input is not taken seriously or visibly acted upon ( Mitchell *et al*., 2024; Taylor *et al*., 2021). More broadly, CYP have similarly characterised their involvement as performative or "tick-box" exercises, with limited opportunity to genuinely influence research processes or outcomes ( Dovey-Pearce *et al*., 2019; Preston *et al*., 2024). Furthermore, marginalised groups—including CYP with complex communication needs and those from ethnic minority or non-English-speaking backgrounds—remain significantly underrepresented in current PPI efforts. Structural barriers such as cultural mismatch, language exclusion, and racism continue to limit meaningful engagement in PPC research ( Mitchell *et al*., 2024).

Secondly, the absence of adequate practical and structural support is another challenge. Research consistently highlights a lack of sustained funding, systemic embedding, and sufficient researcher training as key barriers to implementing youth PPI in a meaningful way ( Hilverda *et al*., 2025). While emotional challenges associated with PPI are less directly addressed in this study, broader PPC literature indicates the critical need for age-appropriate emotional support mechanisms ( National Hospice and Palliative Care Organization, 2022; Taylor *et al*., 2021). The need for flexible and personalized communication approaches, given young people’s widely varying preferences, was also highlighted ( Taylor *et al*., 2021). Similarly, across broader health research, researchers often lack the training and structural support necessary to involve CYP in ways that are responsive to their diverse preferences and engagement needs ( Rouncefield-Swales *et al*., 2021), a challenge that is heightened by the relational and emotional complexities inherent in PPC ( Mitchell *et al*., 2019; Mitchell *et al*., 2021).

Thirdly, ethical and relational concerns remain insufficiently addressed in PCC research. Core principles such as genuine informed consent, careful consideration of emotional vulnerability, and awareness of power dynamics are often only superficially considered. For instance, while a dedicated “ethical issues” discussion was incorporated in a school-based YPAG project ( Roach *et al*., 2021), the assessment of participants’ preferences relied solely on unstructured verbal feedback, missing an opportunity for a more systematic measurement of emotional burden or ethical experience. The crucial need for reflexivity and sensitivity to power imbalances in engaging CYP has been identified in the literature ( Mitchell *et al*., 2019). While established tools such as GRIPP2 and Public Involvement Impact Assessment Framework (PiiAF) offer structured guidance for reporting PPI ( Staniszewska *et al*., 2017), their application within PPC studies remains limited ( Barrett *et al*., 2024).

Although individual studies have explored PPI with CYP in PPC, the existing evidence base is fragmented and lacks comprehensive synthesis. The full extent of young people’s involvement across different research stages - from co-generating ideas and designing research tools to knowledge translation and impact assessment - remains unclear. Likewise, the levels of involvement (e.g., consultation, collaboration, or child-led participation) and the effectiveness of different engagement methods are not well-documented in a systematic way. A systematic mapping of these levels and stages of involvement is therefore critical to understanding both the depth and timing of CYP contributions in PPC research.

This scoping review aims to address these gaps by providing a comprehensive synthesis of the existing literature on the meaningful involvement of CYP as active PPI contributors in paediatric palliative care research. We will illuminate effective practices, highlight common challenges, and identify critical knowledge gaps to inform the development of more inclusive and ethically grounded models for future research. Specifically, we will:

1. Identify and map the various types, levels, and stages of CYP involvement in paediatric palliative care research, including the specific approaches used to facilitate their participation as PPI contributors.
2. Critically examine and categorise the reported outcomes and impacts of CYP involvement in PPI, focusing on its effects on the research process, on CYP themselves, and on ethical and emotional considerations.
3. Investigate the practical and ethical factors that enable or hinder meaningful CYP involvement as PPI contributors in PPC care research.

## Methods

This scoping review will be conducted in accordance with the Joanna Briggs Institute (JBI) guidance for scoping reviews ( Peters *et al*., 2020) and the recommendations for the extraction, analysis, and presentation of results in scoping reviews ( Pollock *et al*., 2023). The review will be reported using the Preferred Reporting Items for Systematic Reviews and Meta-Analyses Extension for Scoping Reviews (PRISMA-ScR) guidelines ( Tricco *et al*., 2018).

### Eligibility criteria

Following the JBI methodological framework for scoping reviews ( Peters *et al*., 2020), this review’s inclusion and exclusion criteria are defined by the Population, Concept and Context (PCC) screening criteria ( Table 1).

**Table 1.** Inclusion criteria and Exclusion criteria.

	Inclusion criteria	Exclusion Criteria
Population	Studies that involve children and/or young people aged 4–24 years of all genders. If adults and/or family members are also included, the study must clearly separate and report data for the 4–24 age group to be eligible.	Studies that focus solely on adults without any relevance or application to paediatric populations. Studies where primary focus is exclusively on parents/ families (or proxy-reported involvement) with no explicit demonstration of the child/young person’s direct involvement. Studies that present combined results for adults (24 + years), parents/families and children/young people without a clear and distinct delineation of findings specific to children and young people aged 4–24 years.
Concept	Report on, evaluate, or describe occurrences or processes of Public and Patient Involvement (PPI) or equivalent terms (e.g., co-production, patient engagement, user involvement, participatory research) in the context of research. Focus on how children and young people are involved in any stage of the research cycle (e.g., priority setting, study design, data collection, analysis, dissemination, knowledge translation). Methods of involvement details the specific participatory methods used to engage children/young people (e.g., focus groups, workshops).	Report on child or young person involvement in clinical care or service delivery only, without a link to research. Focus on child or young person recruitment to research studies, as research subjects or participants, rather than their active involvement as partners in the research process.
Context	Be specifically situated within the context of paediatric palliative care in any setting, including: Specialist PPC services, generalist PPC provision, or care clearly identified as palliative in intent; Children and young people with life-limiting or life-threatening conditions; PPC- relevant aspects such as symptom management, quality of life, end-of-life care, advance care planning, or holistic family support.	Are clearly outside of paediatric palliative care, such as studies focused solely on curative treatments, chronic disease management, or general paediatric research without a palliative care component.
Study Design	Empirical studies (i.e., qualitative, quantitative, mixed methods). Methodological papers discussing approaches to PPI with this population/context, including case studies.	Research protocols (unless they report findings of a completed study).
Publication Type	Published in peer-reviewed journals and in English.	Books, book chapters, opinion pieces, editorials, letters, dissertations/theses, conference abstracts, conference proceedings, toolkits, guidelines, manuals, websites or policy documents.

### Population

This scoping review will include studies involving children and young people (CYP) aged 4–24 years. This age range aligns with previous scoping reviews on youth engagement ( Rouncefield-Swales *et al*., 2021) and encompasses the developmental span from early childhood through adolescence to young adulthood. According to the World Health Organization (WHO), children are defined as under 18, with young people covering the age range 10–24 ( WHO, 2014; Rouncefield-Swales *et al*., 2021), and we use a consistent range of 4–24 to accommodate the diverse age classifications used across studies ( Mitchell *et al*., 2021; Rouncefield-Swales *et al*., 2021; Taylor *et al*., 2021). Detailed characteristics of the CYP participants, such as age, gender, and condition, will be collected during data extraction.

### Concept

This review focuses on Public and Patient Involvement (PPI), which we define as the active and meaningful participation of CYP in the research process beyond their traditional role of research subjects. Drawing from the INVOLVE definition, this includes research conducted “with” or “by” members of the public, rather than “to,” “about,” or “for” them ( INVOLVE, 2012). PPI involves engaging participants in a genuine way across all phases—planning, design, implementation, analysis, and reporting—rather than treating them solely as data sources ( Hoddinott *et al*., 2018; Mitchell *et al*., 2019). For this scoping review, we will include only those studies that explicitly describe or evaluate CYP involvement as PPI contributors in PPC research. Examples of such involvement include co-design workshops, youth advisory groups, structured consultation processes, or the development of participatory framework ( Mitchell *et al*., 2021; Mitchell *et al*., 2024; Rouncefield-Swales *et al*., 2021).

### Context

The context for this review is paediatric palliative care (PPC) research. We will include studies conducted in any PPC settings, including hospitals, hospices, or community-based services. For this review, paediatric palliative care (PPC) is defined as the active, holistic care of a child’s physical, psychological, sociocultural, and spiritual needs—and comprehensive support for the family—from the moment of diagnosis and throughout the illness trajectory, regardless of whether curative treatments are pursued ( Fraser *et al*., 2021; WHO, 2023).

PPC research spans the development, implementation, and evaluation of approaches aimed at improving care experiences and outcomes, such as symptom management, communication, decision-making, and service delivery ( Himelstein *et al*., 2004; Nelson *et al*., 2018). While some studies may not explicitly use the term ‘palliative care’, they will be included if their scope clearly aligns with PPC principles—such as end-of-life support, bereavement care, or family-centred interventions for life-limiting illnesses. Studies focused solely on curative treatments or chronic disease management without a palliative care component will be excluded.

### Search strategy

Our search strategy will follow the three-step process outlined by the Joanna Briggs Institute ( Peters *et al*., 2020). In the first step, we conducted a preliminary limited search in two databases, EMBASE (Ovid) and CINAHL (EBSCO). The purpose of this initial step was to identify a broad range of relevant articles and to analyse their titles, abstracts, and indexing terms. This analysis allowed us to identify key keywords and subject headings (e.g., Emtree terms in Embase) commonly used in the literature on patient and public involvement (PPI) in pediatric palliative care. The insights from this preliminary search were used to inform and refine our comprehensive search strategy.

For the second step, a comprehensive and systematic search will be conducted across the following electronic databases: EMBASE (Ovid), PubMed, CINAHL (EBSCO), Scopus and the Cochrane Library. To maximise relevance and completeness, the search strategies will be precisely adapted to the unique syntax and indexing systems of each database.

A specialist librarian at Dublin City University (DCU) was consulted in the development of our search strategy ( Table 2). Their expert input will continue to guide the refinement of our search terms and database syntax. We will maintain close collaboration with the librarian throughout the data collection phase to ensure consistency and comprehensiveness in all searches.

**Table 2.** Sample search strategy.

Search ID	Search Terms
S1	child'/exp OR adolescent'/exp OR 'young adult'/exp OR 'pediatrics'/exp
S2	patient participation'/exp OR 'consumer participation'/exp OR 'patient engagement'/exp OR 'stakeholder participation'/exp
S3	palliative therapy'/exp OR 'terminal care'/exp OR 'hospice care'/exp OR 'terminally ill patient'/exp
S4	pediatric OR paediatric OR child* OR adolescen* OR teen* OR "young people" OR "young adult*" OR CYP
S5	"patient and public involvement" OR PPI OR PPIE OR involve* OR engagement OR participation OR "public engagement" OR "patient engagement" OR "user engagement" OR "patient participation" OR "stakeholder involvement" OR "knowledge user" OR co-creat* OR co-produc* OR co-design OR codesign OR coproduction OR co-engagement OR "participatory research"
S6	palliat* OR "hospice care" OR "end of life" OR "end of life care" OR "terminal care" OR "terminally ill" OR "compassionate care" OR "comfort care" OR "supportive care" OR PPC OR "palliative approach"
S7	S1 OR S4
S8	S2 OR S5
S9	S3 OR S6
S10	S7 AND S8 AND S9

### Study selection

All search results will be exported into Zotero, a bibliographic reference manager, for centralized citation management. The deduplicated set of references will then be imported into Covidence, a systematic review platform, to facilitate the screening and data management process.

Study selection will be conducted in two stages using Covidence. In the first stage (title and abstract screening), two reviewers (AM, RS) will independently screen all titles and abstracts against the predefined inclusion criteria. Any discrepancies will be resolved by consensus or, if needed, by discussion with a third reviewer (VL). For the second stage (full-text review), the same two reviewers (AM, RS) will independently evaluate full-text articles against the eligibility criteria. Disagreements at this stage will also be resolved by consensus or, if necessary, adjudication by a third reviewer (VL). A detailed log of studies excluded during full-text review, along with the reasons for their exclusion, will be maintained and reported in a PRISMA-ScR flowchart.

### Data extraction

A structured data charting template will be developed to guide the extraction process. One reviewer (AM) will perform the initial data charting, and the form will be piloted on a subset of included studies to ensure clarity and consistency. The extracted data will be cross-checked by a second reviewer (RS) for accuracy and completeness. Any discrepancies will be resolved by a third reviewer (VL).

The extracted data will include the following elements, categorised to align with the review objectives:

**(a) Study characteristics**

- Citation Details: Author(s), title, year of publication, country, and study setting.
- Study Design: Specific methodology used (e.g., qualitative, quantitative, mixed-methods).
- CYP Participant Characteristics: Age, health condition, gender, education status, ethnicity/cultural background, geographical location, language/communication needs, and disability status.

**(b) PPI activity**

- Type of Involvement: Specific PPI activities described (e.g., workshops, advisory groups, co-design sessions).
- Level of Participation: Classified using established typologies:
  - Lansdown’s typology ( Lansdown, 2001): Consultation, Collaboration, Child-led.
  - Shier’s framework ( Shier, 2001): Opportunities and Obligations dimensions to capture structural supports (e.g., language aids, training, debriefing).
- Stage of Research: Mapped to the IUA/Campus Engage Engaged Research Framework (2022), including key phases such as Co-Generating Ideas, Research Planning and Design, Data Collection, Analysis, and Knowledge Mobilization.
- Recruitment Strategies: How CYP were recruited (e.g., open calls, existing networks) and whether diversity or inclusion strategies were reported.

**(c) Outcomes and impacts of PPI**

- Reported Outcomes: Outcomes will be mapped onto the three domains of the Public Involvement Impact Assessment Framework (PiiAF) ( Popay *et al.,* 2014):
  - Methodological Improvements: For example, youth-friendly materials, co-developed data tools.
  - Direct Impacts on CYP: For example, enhanced self-efficacy, communication skills.
  - Ethical and Emotional Reflections: For example, use of pre-/post-engagement support, debriefing processes.

**(d) Barriers and facilitators**

- Factors: Barriers and facilitators will be categorized into:
  - Practical/Logistical: Accessibility, timing, resources.
  - Relational/Interpersonal: Trust, communication, power dynamics.
  - Researcher/Organizational Capacity: Researcher training, institutional infrastructure.
  - Developmental: Age, cognitive stage, communication needs.
  - Contextual/Systemic: Broader ethical or cultural limitations.

**(e) PPI reporting quality**

- Reporting Guidelines: We will assess if studies explicitly used recognized reporting guidelines (e.g., GRIPP2).
- RAG Rating System: A simple Red–Amber–Green (RAG) rating system will be applied to evaluate the comprehensiveness of PPI reporting across six domains ( Preston *et al*., 2023):
  - Red (Unmet): No evidence reported.
  - Amber (Partially met): Some mention with limited detail.
  - Green (Fully met): Clear explanation and examples provided.

Modifications to the data extraction instrument, if necessary, will be collectively reviewed and resolved through detailed discussion among reviewers prior to individual use. All extracted data will be stored, managed, and exported to Excel for further analysis.

### Patient and Public Involvement strategy for this scoping review

We will collaborate with a panel of 3–5 young people (aged 18–24) throughout this scoping review. This collaboration aims to ensure their perspectives are central to the study's direction and outputs. We will recruit young people through established youth engagement networks (e.g., Barretstown, university groups) and aim for diverse representation across backgrounds and experience. Engagement will be flexible and accessible, using a blend of online workshops and asynchronous feedback methods such as collaborative documents and voice notes. All sessions will be co-facilitated by trained researchers and use youth-friendly, visually engaging materials.

Our collaboration with the youth panel will follow a four stage: approach

(1) Following protocol submission, a workshop will be held to introduce the project and gather early-stage input. We will discuss participants' prior experiences with research, explore what aspects of youth perspectives are often underrepresented, and refine the review's language and framing based on their suggestions.

(2) After data extraction, we will hold a second workshop to collaboratively interpret the findings. Participants will review a plain-language summary of the extracted data and provide feedback on preliminary themes. This ensures their lived experience informs the thematic synthesis, positioning them as co-analysts.

(3) In a final workshop, we will co-produce youth-facing outputs to communicate the review's findings. Participants will choose the format (e.g., summary leaflet, video, blog post) and decide how their voices and contributions are included and explicitly acknowledged. This stage ensures dissemination is accessible and youth-led.

(4) Panel members will be invited to provide feedback on their experience through a choice of methods (e.g., reflective form, one-to-one interview).

Their feedback will assess the quality and impact of their involvement, and will be used to inform a critical reflection section in the final review, contributing to best practices for future youth engagement.

### Collating, summarising and reporting the results

Following the charting process, we will undertake a qualitative content analysis, guided by the Joanna Briggs Institute (JBI) methodology for scoping reviews ( Pollock *et al*., 2023) and drawing on the approach outlined by Elo and Kyngäs (2008). This analytic process will be conducted in three stages: (i) *Preparation*, involving familiarisation with the data and the identification of units of analysis; (ii) *Organisation*, through open coding, categorisation, and the development of themes; and (iii) *Reporting*, where findings will be synthesized and presented in a structured narrative and tabular format.

A deductive coding framework will be applied to map the extracted data against predefined categories, enabling the identification of recurring themes, methodological patterns, and insights related to the involvement of CYP as PPI contributors in PPC research. The analysis will remain open to the identification of new subcategories, allowing for inductive refinement of the coding framework. Regular team discussions will support the iterative development of codes and ensure consistency, reflexivity, and analytical rigour.

Findings will be presented in both tabular and narrative formats, allowing for cross-study comparison of engagement practices, reported outcomes, and ethical, practical, and emotional considerations. Where applicable, frequency counts will be used to quantify the distribution of involvement types, while narrative synthesis will contextualise the implications of different approaches to CYP participation ( Tricco *et al*., 2018).

## Discussion and conclusion

This scoping review will provide a critical and comprehensive synthesis of current practices, reported outcomes, and methodological approaches to involving CYP as active PPI contributors in PPC research. By systematically mapping the extent and nature of youth involvement across the entire research cycle, we will provide key insights into how CYP currently contribute to research design, implementation, and dissemination in ways that are both meaningful and developmentally appropriate ( Mitchell *et al*., 2019; O’Hara *et al*., 2017; Rouncefield-Swales *et al*., 2021).

Our findings are expected to highlight significant gaps within current practice. This includes the inconsistent reporting of PPI activities ( Staniszewska *et al*., 2017) and the underrepresentation of marginalised youth ( Mitchell *et al*., 2024). We anticipate that our review will show that while some studies offer critical ethical reflections, they often fall short by relying solely on unstructured feedback to gauge ethical experiences, failing to systematically document issues like emotional burden ( Roach *et al*., 2021).

Crucially, this review will also illuminate how participatory approaches can fundamentally refine research questions, materials, and methods to better reflect real-world priorities ( Barrett *et al*., 2024). Furthermore, it will demonstrate how early relationship-building activities can significantly boost adolescents’ confidence and willingness to engage in research ( Costello & Dorris, 2020).

In our final review, we will discuss the limitations of both the existing literature and our own scoping review methodology, acknowledging areas where the current evidence base may be incomplete. For example, the fragmented and inconsistently reported nature of the existing literature may prevent a full and comprehensive synthesis of certain practices. Notwithstanding this, by synthesising and critically examining the available evidence, this review will inform future PPI efforts in PPC, establishing principles and strategies that support ethical, inclusive, and truly impactful engagement. Ultimately, this work will play a crucial role in advancing the development of robust participatory models that genuinely value CYP as co-creators of knowledge, thereby enhancing the rigour, relevance, and responsiveness of palliative care research for the benefit of all involved.

## Declarations

### Ethics approval and consent to participate

Ethical approval and consent were not required

## Data Availability

No data is associated with this article. PRISMA-ScR Checklist associated with this article is available on OSF, under the project title “PRISMA-ScR Checklist for Engaging Children and Young People in Pediatric Palliative Care Research: A Scoping Review Protocol of Patient and Public Involvement Practices” available at: https://doi.org/10.17605/OSF.IO/GZ9S2 ( Molati *et al.*, 2025). Data are available under the terms of the Creative Commons Zero "No rights reserved" data waiver (CC0 1.0 Public domain dedication).
