# Supplementary material for: Engaging Children and Young People in Pediatric Palliative Care Research: A Scoping Review Protocol of Patient and Public Involvement Practices
Source: HRB Open Res. 2025 Sep 23;8:107. [Version 1] doi: 10.12688/hrbopenres.14236.1 (PMC12824480; doi:10.12688/hrbopenres.14236.1)
Supplement: Peer review history

# Reviewer report: Reviewer response for version 1

**DOI:** 10.21956/hrbopenres.15657.r52408

**Published:** 2026-01-21

**Authors:** Jane Chudleigh

**Affiliations:** 1 King's College London, London, England, UK

Thank you for submitting this scoping review protocol. It is well written and would appear to be replicable. 

 I only have the following very minor comments/queries: 

 Page 3 – YPAG needs defining

 Page 4 – In the aim and objectives, the abbreviation PPC could be used for consistency

 Page 7 – Will the young people in the PPI group have experience of PPC?

Is the study design appropriate for the research question?

Yes

Is the rationale for, and objectives of, the study clearly described?

Yes

Are sufficient details of the methods provided to allow replication by others?

Yes

Are the datasets clearly presented in a useable and accessible format?

Not applicable

Reviewer Expertise:

Screening, genetics, PPIE

I confirm that I have read this submission and believe that I have an appropriate level of expertise to confirm that it is of an acceptable scientific standard.

# Reviewer report: Reviewer response for version 1

**DOI:** 10.21956/hrbopenres.15657.r50352

**Published:** 2025-10-09

**Authors:** Duncan Randall

**Affiliations:** 1 Department of Nursing Sciences, Bournemouth University, UK

This is a well written protocol from a diverse and experienced team. Given the topic I would like to have seen the involvement of a panel of young people mentioned earlier in the protocol, rather than on page 7 of 9. It would be helpful to have more information on the panel in particular what experience of palliative care the members might have and how would the young people be supported to undertake what for some might be a sensitive or difficult enquiry.

 The search strategy is good but does neglect children living with life threatening conditions and perpetuates a bias in the current research towards more planned palliative care for life limiting conditions. Where the needs of children requiring shorter episode of palliative care in acute settings are not considered. This would also affect perinatal death and neonatal death, it ought to be acknowledged that a large proportion of childhood death occurs in these periods and infancy which of course this review would not address. 

 I understand the age restriction of 4-24 but have to point out that in other disciplines such as education and social work researchers have included younger children using adapted often arts based methods. There ought I think to be consideration of what Pia Christensen termed cultures of communication (Christensen and Prout 2002). That as adults we tend to assume younger children will have poor cognition when in fact it has been shown they have very good understanding. We ought perhaps to take a more critical approach to our own cultures of communication with children in our communities. This also relates to another central problem which is that children living with serious health related suffering ( Knaul et al 2018, Kwete Jaing et al 2024) may have cognitive challenges and communication difficulties related to their health condition this together with the nature of palliative and end of life research raises ethical and moral debates about patient involvement. It would have been helpful for readers I think to have considered the wider social/cultural and political aspects of these debates. An aspect I feel fairly confident that will arise in the scoping review is that of proxy reporting, where children's carers are asked what their child thinks about their care. It would have been good to outline an approach to proxy reporting in the reviewed papers and to set out how this might be assessed. It can be very difficult to determine the degree to which a child is able to give their opinion in a very adult centric world in which they and their carers are reliant on social support and healthcare providers.

 A very minor point the citation for Campus Engaged Research on page 6 ( stage of research under (b) PPI activity) is not consistent with the citation in the end list. It would help a reader to amend this so the citation titles match.

 This is a detailed, well written protocol for a scoping review on what is a very complex topic, having set out the scoping review protocol here I am sure that the authors will be able to expand on some of the debates in their report on the scoping review.

Is the study design appropriate for the research question?

Yes

Is the rationale for, and objectives of, the study clearly described?

Yes

Are sufficient details of the methods provided to allow replication by others?

Yes

Are the datasets clearly presented in a useable and accessible format?

Yes

Reviewer Expertise:

Children's Palliative Nursing Care, Children's Nursing Theory

I confirm that I have read this submission and believe that I have an appropriate level of expertise to confirm that it is of an acceptable scientific standard.

## References

[1] : Alleviating the access abyss in palliative care and pain relief—an imperative of universal health coverage: the Lancet Commission report. *The Lancet* .2018;391(10128) : 10.1016/S0140-6736(17)32513-8 1391-1454 10.1016/S0140-6736(17)32513-8 29032993

[2] : Global Assessment of Palliative Care Need: Serious Health-Related Suffering Measurement Methodology. *Journal of Pain and Symptom Management* .2024;68(2) : 10.1016/j.jpainsymman.2024.03.027 e116-e137 10.1016/j.jpainsymman.2024.03.027 38636816 PMC11253038

[3] : Working with Ethical Symmetry in Social Research with Children. *Childhood* .2002;9(4) : 10.1177/0907568202009004007 477-497 10.1177/0907568202009004007
